# The Immunomodulatory Effects of Mannan on the Fungicidal Activity of Macrophages Against *Candidozyma auris* Bloodstream Infection Associated with the GPCR-PI3K-NF-κB Signaling Pathway

**DOI:** 10.3390/microorganisms14092063

**Published:** 2026-09-16

**Authors:** Rong Wang, Wenqing Liu, Xiaoyu Peng, Shu Gong, Zhangyong Song, Hong Yu

**Affiliations:** 1 School of Basic Medical Sciences, Southwest Medical University, Luzhou 646000, China; 2 Public Center of Experimental Technology, Southwest Medical University, Luzhou 646000, China

**Keywords:** mannan, Ana-1 macrophage, *Candidozyma auris*, killing effect, immune response

## Abstract

*Candidozyma auris* is an emerging multidrug-resistant fungal pathogen, which causes high mortality and nosocomial outbreaks worldwide. Macrophages are key innate immune cells that clear fungi through phagocytosis, reactive oxygen species, and cytokine secretion. Mannan, a major fungal cell wall polysaccharide, has immunomodulatory properties and can interact with receptors such as C-type lectins and Toll-like receptors 2/4, which are associated with nuclear factor kappa-light-chain-enhancer of activated B cells and mitogen-activated protein kinase signaling in macrophages. However, its role in host defense against *C. auris* remains unclear. In this study, the effects of mannan against *C. auris* infection were evaluated using Ana-1 macrophages and murine models. Cell Counting Kit-8 assay, neutral red uptake, and fluorescence microscopy assay showed that mannan with safety concentrations of 0–160 μg/mL significantly increased the phagocytosis activity of Ana-1 macrophages, and following pre-stimulation of Ana-1 macrophages with mannan for 1 h, the phagocytic ability of these macrophages to *C. auris* was significantly enhanced at 30 min, and the killing ability was significantly higher than that of the control group at 4 h. Transcriptomic analysis combined with real-time quantitative PCR and Western blotting showed that mannan enhanced the immune response of Ana-1 macrophages, which was associated with changes in GPCR-related PI3K/NF-κB signaling, and improved their killing activity against *C. auris*. In vivo efficacy was further validated in mice and zebrafish via fungal burden, histopathology, and immune cell recruitment assays, which confirmed that mannan can effectively prevent *C. auris* bloodstream infection. These findings provide insights into mannan’s immunomodulatory mechanisms and support its potential in developing new antifungal immunotherapies for *C. auris* infection.

## 1. Introduction

Invasive fungal infections have become a significant global public health challenge, with *Candidozyma auris* (formerly *Candida auris*) drawing particular attention due to its multidrug resistance, high mortality rate, and potential for nosocomial transmission [1,2,3]. Since its first report in 2009, *C. auris* has caused nosocomial outbreaks in multiple countries worldwide. In 2026, the prevalence of *C. auris* has shown a pattern of multiple outbreaks and global spread, with particularly rapid increases observed in the United States and the United Kingdom, and new cases reported in Canada [4,5,6]. Public healthcare systems in Africa and Europe are similarly under significant pressure [7,8,9]. Moreover, clinical isolates exhibit resistance rates exceeding 90% against frontline antifungal drugs such as fluconazole, and the pathogen can persist on environmental surfaces for extended periods, significantly complicating infection control efforts [2,3,10]. The mortality rate due to *C. auris* infections can reach 40–60%, particularly in immunocompromised individuals, who are highly susceptible to fungal bloodstream infections and multiple organ failure. Currently, echinocandins are the mainstay of clinical treatment [11,12,13]. However, the emergence of resistant echinocandin strains continues to rise, and *C. auris* possesses unique immune evasion mechanisms, such as reducing the expression of β-glucan to impair recognition by host immune cells [14,15]. In addition, the limited ability of echinocandins to penetrate the blood–brain barrier further constrains treatment efficacy, posing a serious challenge to current antifungal strategies [16]. Therefore, the development of novel antifungal approaches is urgently needed to enhance therapeutic outcomes and curb the ongoing spread of drug resistance.

During *C. auris* infection, the host immune system, particularly the innate immune system, plays a crucial defensive role in recognizing and eliminating the pathogen [17]. Among innate immune cells, macrophages serve as key effector cells by utilizing pattern recognition receptors (PRRs) to detect pathogen-associated molecular patterns present in the fungal cell wall, such as mannan, β-glucan, and chitosan, thereby initiating a cascade of immune responses. In the antifungal process, macrophages exert their killing function by phagocytosing pathogens, generating reactive oxygen species (ROS), and secreting pro-inflammatory cytokines such as tumor necrosis factor-α, interleukin (IL-6), and IL-1β [18]. However, *C. auris* can significantly suppress the phagocytic and killing functions of macrophages through strategies such as forming protective biofilms and altering cell wall component exposure, thereby achieving immune evasion and enabling persistent survival and dissemination within the host [19]. In recent years, studies have shown that G protein-coupled receptors (GPCRs) play an important role in regulating macrophage recognition of pathogens, signal transduction, and immune responses [20]. GPCR-mediated signaling not only contributes to the regulation of inflammatory cytokine expression but is also closely associated with enhanced phagocytic activity [21,22]. Therefore, targeting GPCR-related pathways to enhance macrophage function holds promise as a novel strategy to strengthen host defense against *C. auris* and offers new directions for antifungal immunotherapy research.

Mannan, a polysaccharide present in the cell walls of fungi and plants, is a key structural component of fungal cell walls and has attracted significant attention in immunological research [23]. Its immunomodulatory effects primarily occur through binding to host PRRs, such as C-type lectin receptors and Toll-like receptors (TLR2 and TLR4), thereby activating downstream signaling pathways including nuclear factor kappa-light-chain-enhancer of activated B cells (NF-κB) and mitogen-activated protein kinase (MAPK). This leads to the activation of immune cells and enhances their effector functions. In recent years, mannan has demonstrated significant potential in antifungal immunity. It not only acts as a natural immune activator that promotes the activation of macrophages and dendritic cells, thereby enhancing the clearance of pathogens [24], but has also been shown in several studies to exert notable effects against yeast infections. For example, research has found that mannan can enhance the host immune response against *C. albicans* and *C. auris* by promoting phagocytosis and the secretion of inflammatory cytokines, thereby improving antifungal defense [25,26]. In addition, mannan has shown promise as a vaccine adjuvant, with the potential to improve the efficacy of antifungal vaccines [27,28]. The mechanism by which mannan modulates immune cells to exert antifungal effects against *C. auris* remains unclear.

This study aims to systematically investigate the potential mechanisms by which mannan enhances the candidacidal activity of murine Ana-1 macrophages against *C. auris*. Using Ana-1 cells as a model, the effects of mannan on macrophage phagocytic and killing activity, proinflammatory cytokine secretion, and the activation status of relevant signaling pathways were determined to comprehensively elucidate its immunomodulatory effects. In addition, the underlying molecular mechanisms were evaluated. Prophylactic administration of mannan effectively prevents and mitigates *C. auris* infection in mice. Using zebrafish models, mannan was shown to enhance the number and recruitment capacity of innate immune cells under non-toxic conditions, thereby improving host survival and promoting fungal clearance during *C. auris* infection. This research not only provides further insights into the role of mannan in regulating host immunity but also offers a theoretical foundation for its application as part of antifungal therapeutic strategies, contributing to the development of new approaches to address *C. auris* drug resistance.

## 2. Materials and Methods

### 2.1. Fungal Strains, Materials, and Reagents

Ana-1 macrophages were obtained from Zhongqiao Xinzhou (Shanghai, China). RPMI-1640 medium containing L-glutamine and fetal bovine serum (FBS) were purchased from Gibco (Grand Island, NY, USA). Ana-1 macrophages were cultured in RPMI-1640 medium supplemented with 10% FBS and 1% penicillin–streptomycin and maintained at 37 °C in a humidified atmosphere containing 5% CO_2_. No additional differentiation agents were used. The cells were routinely subcultured under the same culture conditions, and low-passage Ana-1 macrophages in good condition were used for all experiments. The Cell Counting Kit-8 (CCK-8) was purchased from APExBIO (Houston, TX, USA), and the antifade mounting medium with DAPI was obtained from Biosharp (Beijing, China). RNA reverse transcription kits and Real-Time Quantitative PCR (RT-qPCR) kits were purchased from TaKaRa (Dalian, China). Mannan was obtained from Sigma-Aldrich (Merck, St. Louis, MO, USA; Cat. No. M861453). Antibodies against GPCR, PI3K, p-PI3K, Akt, p-Akt, P65, and p-P65 were purchased from Immunity (Chengdu, China), and the glyceraldehyde-3-phosphate dehydrogenase (GAPDH) antibody was obtained from China Sanying Biotechnology Co., Ltd. (Wuhan, China).

The *C. auris* strain used in this study was preserved at the Laboratory of Pathogenic Fungi and Disease Control, Southwest Medical University. The strain was cultured on yeast extract peptone dextrose (YPD) medium. Prior to each experiment, a single pre-activated colony from the YPD agar plate was selected and cultured in YPD liquid medium at 37 °C for 16–18 h. Mannan was dissolved in serum-free 1640 medium by heating and stirring thoroughly at 60 °C until fully dissolved, and a stock solution was prepared at a concentration of 0.02 g/mL.

### 2.2. CCK-8 Cell Proliferation Assay

Cell proliferation was assessed using the CCK-8 assay according to the manufacturer’s instructions. Ana-1 cells (1 × 10^5^/mL) were seeded at 100 μL per well in 96-well plates. The treatment groups were supplemented with mannan at final concentrations of 10, 20, 40, 80, and 160 μg/mL, and incubated for 3 h or 5 h. After incubation, the supernatant was removed and cells were washed with phosphate-buffered saline (PBS) 2–3 times. Then, 100 μL of culture medium containing 10% CCK-8 reagent was added, and the cells were incubated at 37 °C for 2 h. Absorbance was measured at 450 nm. Blank wells containing 100 μL of complete culture medium without cells were included on each plate. The absorbance of the blank wells was used for background correction.

### 2.3. Neutral Red Uptake Assay

The neutral red uptake assay was used to evaluate the pinocytic activity of Ana-1 cells according to a previous method [29]. Briefly, Ana-1 cells (1 × 10^6^/mL) were seeded at 100 μL per well in 96-well plates and treated with mannan at concentrations of 10, 40, and 160 μg/mL. After a designated incubation period, the supernatant was discarded and the cells were washed with PBS 2–3 times. A 0.5% neutral red solution was added, and the cells were incubated at 37 °C for 1 h. The dye was then discarded, and cells were washed again with PBS 2–3 times. Cold sterile distilled water was added to lyse the cells, and absorbance was measured at 540 nm. The absorbance values of the blank wells on each plate were used for background correction.

### 2.4. Fluorescence Staining Phagocytosis Assay

Ana-1 cells (1 × 10^5^/mL) were seeded onto coverslips to prepare cell slides. The experimental group was treated with 40 μg/mL mannan. After incubation, the supernatant was removed and cells were washed with PBS 2–3 times. Freshly activated *C. auris* cells were stained with concanavalin A–fluorescein isothiocyanate (ConA-FITC; Sigma-Aldrich) at 37 °C for 30 min, followed by washing with PBS 2–3 times and adjustment to a concentration of 1 × 10^5^ cells/mL. The stained *C. auris* cells were co-incubated with the Ana-1 cell slides. Following incubation, the supernatant was discarded and cells were washed with PBS 2–3 times. The cells were then fixed with 4% paraformaldehyde and mounted with DAPI-containing antifade mounting medium. The slides were observed and photographed under a fluorescence microscope. The slides were examined and photographed using an Olympus BX63 upright fluorescence microscope (Olympus Corporation, Tokyo, Japan) in bright-field and FITC fluorescence imaging modes. FITC fluorescence was detected at an excitation wavelength of 488 nm and an emission wavelength of 525 nm. For each experimental variant, 3–5 randomly selected fields were imaged, and the number of fluorescence-positive cells was quantified.

### 2.5. Collection and Preparation of Samples for Transcriptome Sequencing

Ana-1 cells were seeded in 6-well plates at a concentration of 1 × 10^6^ cells/mL and incubated overnight at 37 °C. The following day, the supernatant was removed, and the experimental group was treated with 2 mL of mannan (40 μg/mL) for 1 h, followed by three PBS washes. An equal volume of *C. auris* suspension (1 × 10^6^ cells/mL) was then added and co-incubated for 30 min. After washing three times with PBS, 2 mL of complete medium was added, and cells were incubated for an additional 4 h before terminating the experiment. Each well was treated with 1 mL of RNAiso Plus reagent. Total RNA was extracted, and samples were stored at −80 °C before being sent on dry ice to Biomarker Technologies (Qingdao, China) for library preparation and next-generation sequencing to analyze mRNA expression profiles. The criteria for determining differential genes were set as follows: significance level (adjusted *p*-value) < 0.05, fold change for upregulation/downregulation > 2.0, differential analysis software: DESeq2 (Version [1.42.0], Bioconductor, Seattle, WA, USA), and *p*-value adjustment method for multiple testing: Benjamini–Hochberg (BH). The transcriptomic data were then uploaded to the NCBI Sequence Read Archive BioSample database under BioProject ID: PRJNA1480400.

### 2.6. RT-qPCR Analysis

To evaluate the effects of mannan on gene transcriptional activity in macrophages phagocytosing *C. auris* and in mouse spleen tissue, both in vitro-cultured Ana-1 cells and mouse spleen samples were collected. Total RNA was extracted using RNAiso reagent (Takara), and RNA concentration was measured using a NanoDrop One spectrophotometer (Thermo Fisher Scientific, Shanghai, China). cDNA synthesis was performed using a reverse transcription kit (Takara), followed by RT-qPCR using a SYBR Green-based detection system on a 7500 Fast Real-Time PCR System (Thermo Fisher Scientific). A two-step RT-qPCR protocol was performed for 40 amplification cycles, followed by melting curve analysis to evaluate the specificity of the amplification products. The primer pairs used in this study were derived from previously established sequences in our laboratory and were used for target gene expression analysis. The specificity of each primer pair was confirmed by melting curve analysis, and all primer pairs generated a single specific melting peak without detectable non-specific amplification products or primer-dimer formation. Primer sequences are listed in Table A1, and *GAPDH* was used as the internal control. Relative gene expression levels were calculated using the 2^−ΔΔCT^ method [30].

### 2.7. Western Blot Analysis

Total protein was extracted from cells lysed with Radio Immunoprecipitation Assay buffer. The protein concentration of each sample was determined using a bicinchoninic acid (BCA) protein assay, and equal amounts of total protein were loaded for electrophoresis. Protein samples were separated by 10% sodium dodecyl sulfate-polyacrylamide gel electrophoresis and transferred onto polyvinylidene difluoride membranes. The membranes were blocked with 5% Bovine Serum Albumin (BSA) and incubated overnight at 4 °C with primary antibodies (1:1000). The following day, the membranes were incubated with Horseradish Peroxidase-Streptavidin-conjugated goat anti-rabbit IgG secondary antibodies (1:4000) at room temperature for 2 h. Protein bands were visualized using enhanced chemiluminescence (ECL) reagents and detected using a Tanon 5200CE Chemiluminescence Imaging System (Tanon Science & Technology Co., Ltd., Shanghai, China). GAPDH was used as the loading control. Band intensities were quantified using ImageJ 1.53 software by densitometric analysis, and no specialized plugin was used for image analysis.

### 2.8. Molecular Docking Analysis

Molecular docking was performed to investigate the interactions between the ligand (mannan) and the GPCR [31]. Initially, AutoDock 4.2.6 Tools were employed for the preparation of ligands and receptors. This included the addition of polar hydrogen atoms, assignment of Gasteiger charges, definition of rotatable bonds, and specification of the docking grid around potential binding sites. Configuration files suitable for AutoDock Vina were subsequently generated. Docking simulations were carried out using AutoDock Vina. Multiple rounds of local search and iterative optimization were conducted to identify the optimal docking conformations with the lowest binding energies. The resulting docking poses were further analyzed and visualized using PyMOL 3.0.3 and Maestro 14.3 software. Key binding residues and hydrogen bond interactions were identified and illustrated, enabling the evaluation of binding stability and the potential mechanisms of ligand–receptor interaction.

### 2.9. Mouse Models, Whole Blood Cell Analysis, and Histochemical Staining

Female and male BALB/c mice (7–8 weeks old, weighing 20–25 g) were purchased from Chongqing Tengxin Biotechnology Co., Ltd. (Chongqing, China). All animal experiments were approved by the Laboratory Animal Ethics Committee of Southwest Medical University (Approval No. 20220817-016). A *C. auris* bloodstream infection model was established according to a previous method [32]. Briefly, in the prophylactic model, mice were randomly divided into five groups (*n* = 8 per group) as follows: (1) Control group: administered 100 μL of sterile normal saline via oral gavage for 3 days, followed by a tail vein injection of 100 μL sterile saline on day 3; (2) Model group: administered 100 μL of sterile normal saline via oral gavage for 3 days, followed by a tail vein injection of *C. auris* at 1 × 10^7^ Colony-Forming Units (CFU)/mL on day 3; (3) 150 mg/kg mannan group: administered 150 mg/kg mannan via oral gavage for 3 days, followed by *C. auris* infection on day 3; (4) 250 mg/kg mannan group: same as above, with 250 mg/kg mannan administered; (5) Isavuconazole (ISA) group: administered 110 mg/kg isavuconazole via oral gavage three times daily for 3 days, followed by *C. auris* injection on day 3. Blood was collected from the retro-orbital plexus and preserved in EDTA anticoagulation tubes for whole-blood cell analysis. One-third of the liver, kidney, spleen, lung, and brain tissues were used to assess fungal burden. Another one-third of these tissues was fixed in 4% paraformaldehyde and embedded in paraffin. Liver tissue sections were stained with hematoxylin and eosin (H&E), and kidney, small intestine, and large intestine sections were stained using the periodic acid-Schiff (PAS) method for histopathological examination. The remaining third of each organ sample was snap-frozen in liquid nitrogen for subsequent cytokine detection.

### 2.10. Observation of Macrophage and Neutrophil Recruitment in the Zebrafish Model

Adult SZ5 and SZ59 zebrafish (two males and two females) were obtained from the Zebrafish Technology Platform of Southwest Medical University. All zebrafish experiments were covered by the same ethical approval from the Laboratory Animal Ethics Committee of Southwest Medical University (Approval No. 20220817-016). The SZ5 line carries a macrophage-specific fluorescent reporter driven by the mpeg1 promoter, allowing specific visualization of macrophages, whereas the SZ59 line expresses the red fluorescent protein DsRed2 under the control of the lyz promoter, enabling specific visualization of neutrophils. They were maintained in a recirculating aquaculture system under separate housing by sex at 27 ± 2 °C, salinity of 450–550 µS/cm, and pH 7–8, with a 14-h light/10-h dark cycle. The fish were fed brine shrimp twice daily. On the evening before the experiment, the male and female fish were placed in spawning tanks at a 2:1 ratio with a transparent divider. At 8:00 a.m. the next morning, the divider was removed, and transparent, glossy embryos were collected and transferred to circulating water, then incubated in an incubator (28 °C, 12 h light/12 h dark cycle). Residual debris was removed daily and one-third of the water was refreshed. At 24 h post-fertilization (hpf), embryos were transferred to embryo medium containing 0.025% 1-phenyl 2-thiourea (PTU) to inhibit melanin synthesis. From day 3 onward, larvae were fed egg yolk. At 72 hpf, larvae were randomly divided into five groups (30 larvae per group): Blank, Model (infection only), and three mannan-treated groups (60, 120, and 240 mg/L). Each treatment group was incubated in its respective solution for 24 h, while the Blank and Model groups were maintained in an equal volume of rearing water. Following incubation, toxicity signs were observed, and the numbers of macrophages and neutrophils were recorded using both light microscopy and an Olympus stereoscopic 3D fluorescence imaging system (Olympus, Tokyo, Japan). Fluorescence images were analyzed using ImageJ software, and the resulting data were statistically analyzed using GraphPad Prism software.

With the exception of the Blank group, larvae from all other groups were microinjected in the yolk sac with 1 nL of *C. auris* suspension (1 × 10^8^ CFU/mL in saline containing 10% phenol red); the Blank group received an equal volume of 10% phenol red diluted in saline. At 12, 36, and 72 h post-infection, immune cell recruitment was observed and recorded using a 3D fluorescence imaging system. At each time point, 10 larvae were randomly selected for fungal burden assessment. In addition, larval survival was monitored at 12, 24, 36, 48, 60, and 72 h post-infection. Mortality time and numbers were recorded to evaluate the protective effect of mannan against infection.

### 2.11. Statistical Analyses

Each experiment was conducted with three independent replicates. Before statistical analysis, data distribution was assessed using the Shapiro–Wilk normality test. Data were considered normally distributed when *p* > 0.05. Statistical analyses were conducted by one-way ANOVA using GraphPad Prism 9 (GraphPad Software Inc., La Jolla, CA, USA). The results represent the average of three independent experiments ± standard deviations (SD). *p*-values < 0.05 were considered significant.

## 3. Results

### 3.1. Mannan Enhances the Phagocytic and Fungicidal Activity of Ana-1 Macrophages Against C. auris at Non-Toxic Concentrations

**Figure 1:**
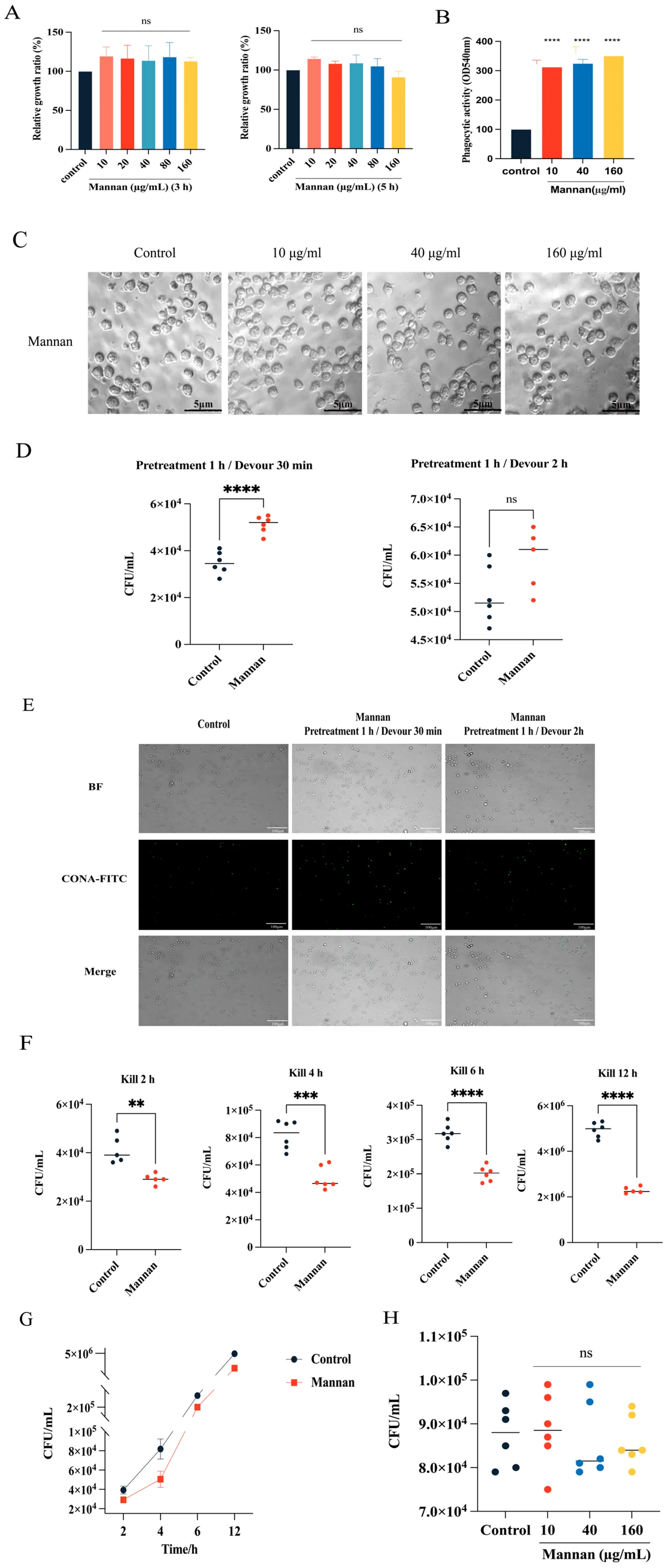
The effect of mannan on the phagocytic and cytotoxic abilities of Ana-1 cells against *Candidozyma auris*. (**A**) The CCK8 method was used to evaluate the cell survival rate following coculture of mannan and Ana-1 cells for 3 h and 5 h. (**B**) A neutral red phagocytosis assay was used to detect changes in the phagocytic activity of Ana-1 cells stimulated with mannan. (**C**) Microscopy was used to observe the morphological changes in Ana-1 cells after stimulation with mannan. Scale bar: 5 μm. (**D**,**E**) Colony counting and fluorescence staining experiments were performed to detect the ability of Ana-1 cells to be prestimulated with mannan for 1 h and phagocytose *C. auris* for 30 min and 2 h. Scale bar: 100 μm. (**F**,**G**) When Ana-1 cells were prestimulated with mannan for 1 h and phagocytosed with *C. auris* for 30 min, their ability to kill *C. auris* was affected at 2 h, 4 h, 6 h, and 12 h. (**H**) Mannan was cocultured with *C. auris* for 4 h, and the results revealed its inhibitory effect on *C. auris*. ns: indicates not significant; ** indicates *p* < 0.01; *** indicates *p* < 0.001; ****: *p* < 0.0001.

To evaluate the effects of mannan on the biological behavior of the murine macrophage cell line Ana-1, its influence on cell proliferation and cytotoxicity at various concentrations was first assessed. Ana-1 cells were co-cultured with mannan at concentrations ranging from 10 to 160 μg/mL for 3 or 5 h. The results showed no apparent cytotoxicity at any concentration or time point (Figure 1A), indicating that mannan is biocompatible within this concentration range and can be considered non-toxic. Based on these findings, the effect of mannan on the phagocytic function of Ana-1 cells using the neutral red uptake assay was further investigated. Mannan significantly enhanced the phagocytic capacity of Ana-1 cells at concentrations of 10, 40, and 160 μg/mL (Figure 1B). Microscopic observation revealed no obvious morphological changes in treated cells (Figure 1C), suggesting that mannan effectively enhances macrophage phagocytic function without inducing cellular abnormalities. To determine whether mannan also promotes the phagocytosis of pathogens, fluorescence staining and Colony-Forming Units (CFU) counting methods were used to evaluate Ana-1 macrophage phagocytosis of *C. auris*. Following pretreatment with 40 μg/mL mannan for 1 h, Ana-1 cells showed significantly increased phagocytosis of *C. auris* at 30 min post-exposure compared to the untreated control group (Figure 1D,E). However, there was no significant difference at 2 h, suggesting that mannan’s enhancement of phagocytic activity primarily occurs during the early phase of phagocytosis. To assess whether mannan also enhances the fungicidal capacity of macrophages against *C. auris*, CFUs at multiple time points were measured. The results showed that Ana-1 cells pretreated with 40 μg/mL mannan for 1 h exhibited significantly increased killing of *C. auris* after 30 min of phagocytosis followed by 2, 4, 6, and 12 h of incubation, with the most pronounced difference observed at 4 h (Figure 1F,G). To rule out the possibility that mannan directly inhibits fungal growth, *C. auris* was incubated with mannan (10, 40, and 160 μg/mL) for 4 h in the absence of macrophages. No significant inhibition of fungal growth was observed under these experimental conditions, suggesting that the enhanced antifungal activity was primarily associated with improved macrophage-mediated killing rather than a detectable direct antifungal effect of mannan (Figure 1H).

### 3.2. Transcriptome Sequencing Results of Mannan Enhancing Ana-1 Cell Killing Ability Against C. auris

**Figure 2:**
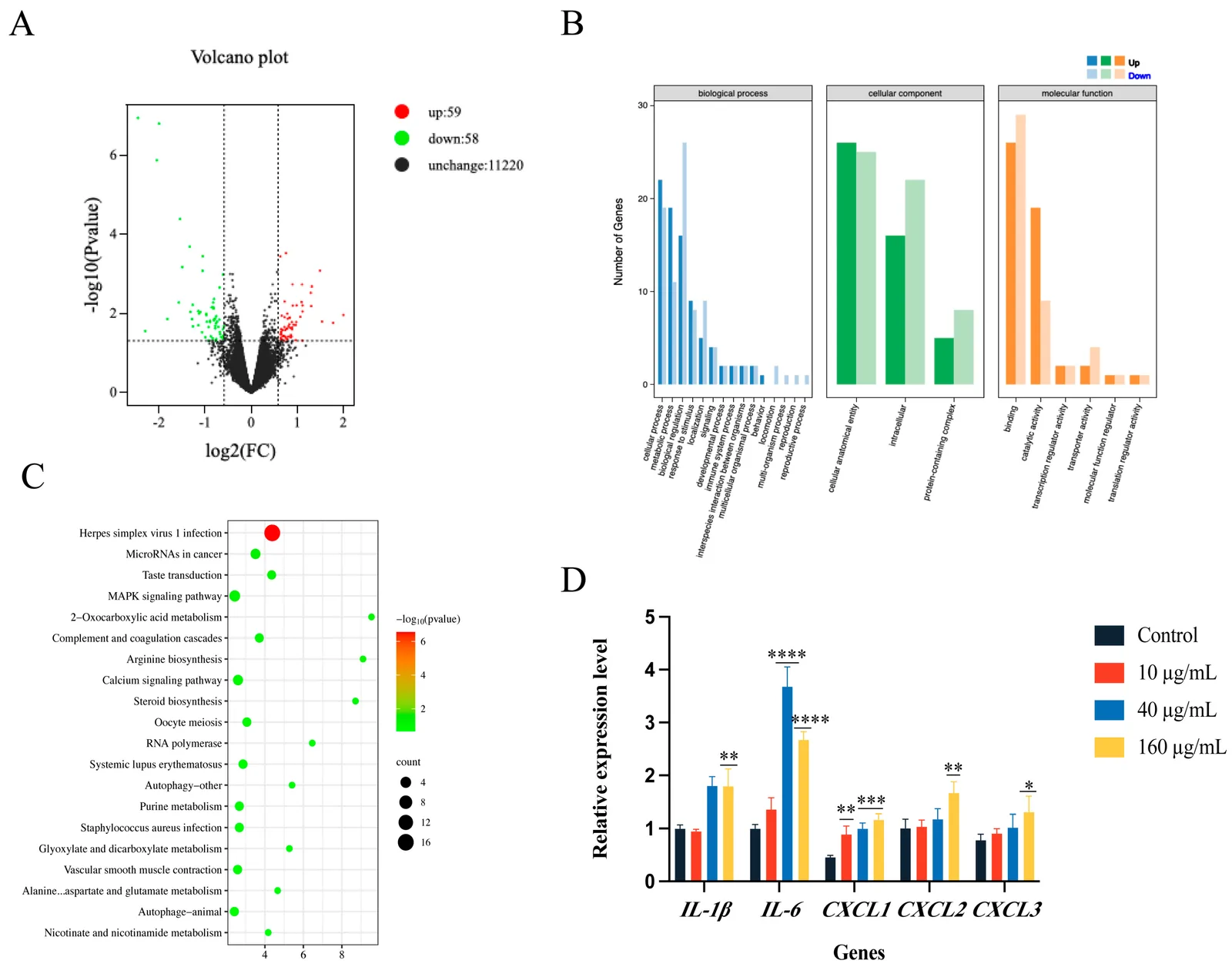
Transcriptomic characteristics of mannan regulating the immune response in Ana-1 cells against *Candidozyma auris*. Following mannan stimulation of Ana-1 cells for 1 h, *C. auris* was phagocytosed for 30 min and killed for 4 h, respectively. (**A**) Volcano diagram of differentially expressed genes (DEGs); each point in the diagram represents a gene; green: downregulated genes; red: upregulated genes. (**B**) Gene Ontology classification diagram of DEGs. (**C**) Bubble diagram of the Kyoto Encyclopedia of Genes and Genomes pathway enrichment analysis of DEGs. (**D**) RT–qPCR detection of the gene expression levels of the inflammatory cytokines *IL-1β*, *IL-6*, and the chemokine *CXCL1-3*. Statistical comparisons were performed between each experimental group and the control group. ns: indicates not significant; * indicates *p* < 0.05; ** indicates *p* < 0.01; *** indicates *p* < 0.001; ****: *p* < 0.0001.

To further explore the underlying molecular mechanisms of the fungicidal activity of Ana-1 macrophages against *C. auris*, transcriptome sequencing analysis of mannan-stimulated Ana-1 cell was performed. A total of 117 genes were identified as significantly differentially expressed, 59 were upregulated and 58 were downregulated. Subsequently, an online bioinformatics platform was used to create a volcano plot of the differentially expressed genes (DEGs) in order to visualize the expression changes (Figure 2A). To clarify the potential biological functions of these DEGs, Gene Ontology (GO) annotation analysis was performed. The results showed that the DEGs were mainly enriched in 24 GO terms, with the majority belonging to biological processes, including 15 terms related to cellular processes, metabolic processes, biological regulation, stress response, and signal transduction. Molecular function terms included six categories involving binding and catalytic activity, while cellular components contained three categories (Figure 2B), suggesting that these DEGs may participate in regulating multiple immune-related biological processes.

Furthermore, Kyoto Encyclopedia of Genes and Genomes (KEGG) pathway enrichment analysis revealed that these DEGs were significantly enriched in several classical signaling pathways, particularly the tumor necrosis factor (TNF) signaling pathway and calcium signaling pathway, both closely associated with inflammation and immune responses (Figure 2C). To validate the transcriptome data and further clarify expression changes of key genes, representative immune regulatory genes were selected for RT-qPCR verification, including inflammatory cytokines *IL-6*, *IL-1β*, and the chemokine *CXCL2*. The results showed significant upregulation of these genes in the mannan-treated group (Figure 2D), consistent with the transcriptome findings.

### 3.3. Mannan Enhances Macrophage Anti-C. auris Activity by Activating the GPCR-PI3K-NF-κB Signaling Pathway

**Figure 3:**
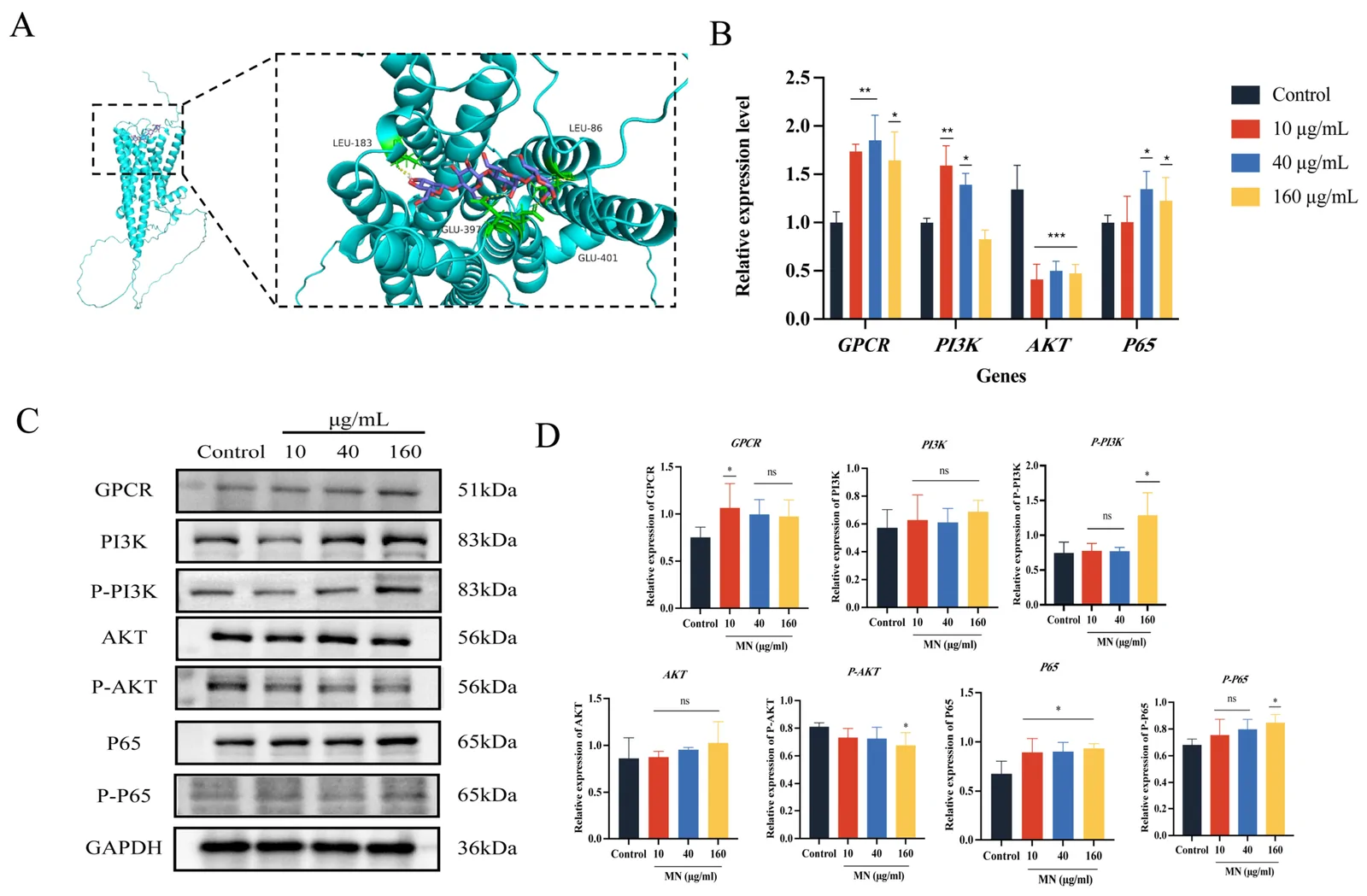
Mannan promotes antifungal responses in Ana-1 macrophages through activation of the GPCR–PI3K–Akt–NF-κB signaling pathway. (**A**) Interaction plot between mannan and GPCR proteins. It is generally believed that a docking energy value less than −4.25 kcal/mol indicates a certain binding activity between the two, a value less than −5.0 kcal/mol indicates good binding activity, and a value less than −7.0 kcal/mol indicates strong binding activity. (**B**) Changes in the mRNA expression of *GPCR*, PI3K, Akt, and NF-kB p65 in Ana-1 cells. (**C**,**D**) Changes in the protein expression of *GPCR*, PI3K, Akt, and NF-kB p65. ns: indicates not significant; * indicates *p* < 0.05; ** indicates *p* < 0.01; *** indicates *p* < 0.001.

To better understand the molecular mechanisms underlying mannan-enhanced fungicidal activity of Ana-1 macrophages against *C. auris*, a comprehensive transcriptomic analysis was conducted. Notably, genes encoding membrane proteins of the GPCR family displayed an upward trend in expression within the PI3K signaling pathway. Although these transcriptional changes did not reach conventional thresholds for statistical significance, the observed upregulation pattern prompted us to further investigate the potential involvement of GPCR-related signaling in the immunomodulatory effects of mannan. The role of GPCR-mediated PI3K/Akt signaling in diverse pathological and immunological contexts is well-established and includes cancer, diabetes, primary immunodeficiencies, and inflammatory diseases [33]. Based on these observations, we hypothesized that GPCR-related signaling may represent an upstream regulatory component of the immunomodulatory effects of mannan. To investigate whether mannan might directly interact with GPCR proteins, molecular docking analysis was conducted (Figure 3A). The results revealed a binding energy of −7.8 kcal/mol between mannan and GPCR, suggesting a favorable predicted interaction between mannan and the candidate GPCR protein. Although molecular docking cannot establish direct binding in a biological system, this result provides computational evidence supporting the possibility of a potential interaction between mannan and GPCR. Combined with the previous RT-qPCR results (Figure 2D), significant upregulation of multiple key downstream effectors in the PI3K signaling pathway, such as *IL-6*, *IL-1β*, and *CXCL2*, were observed, providing additional evidence that PI3K-related signaling may contribute to the immunomodulatory effects of mannan.

To validate this hypothesis, GPCR and its downstream classical antifungal signaling pathways were investigated. RT-qPCR analysis showed that after mannan treatment of Ana-1 cells followed by co-incubation with *C. auris*, the mRNA levels of GPCR, PI3K, and NF-κB p65 were significantly upregulated, whereas Akt expression was downregulated (Figure 3B). Furthermore, Western blot analysis of protein expression levels confirmed marked increases in GPCR, PI3K, and NF-κB p65 proteins, while Akt protein expression showed a decreasing trend (Figure 3C,D). Importantly, the consistent changes observed at both the mRNA and protein levels provide independent experimental support for the involvement of GPCR-related signaling and its downstream PI3K/NF-κB pathway, despite the lack of statistical significance for the initial GPCR transcriptional changes identified by transcriptomic analysis. In summary, these results support a model in which mannan may enhance macrophage phagocytosis and the killing of *C. auris* through GPCR-related signaling and subsequent activation of the PI3K/NF-κB pathway, thereby promoting the expression of inflammatory cytokines and chemokines.

### 3.4. Immunoprotective Effect of Mannan in a Mouse Model of C. auris Bloodstream Infection

**Figure 4:**
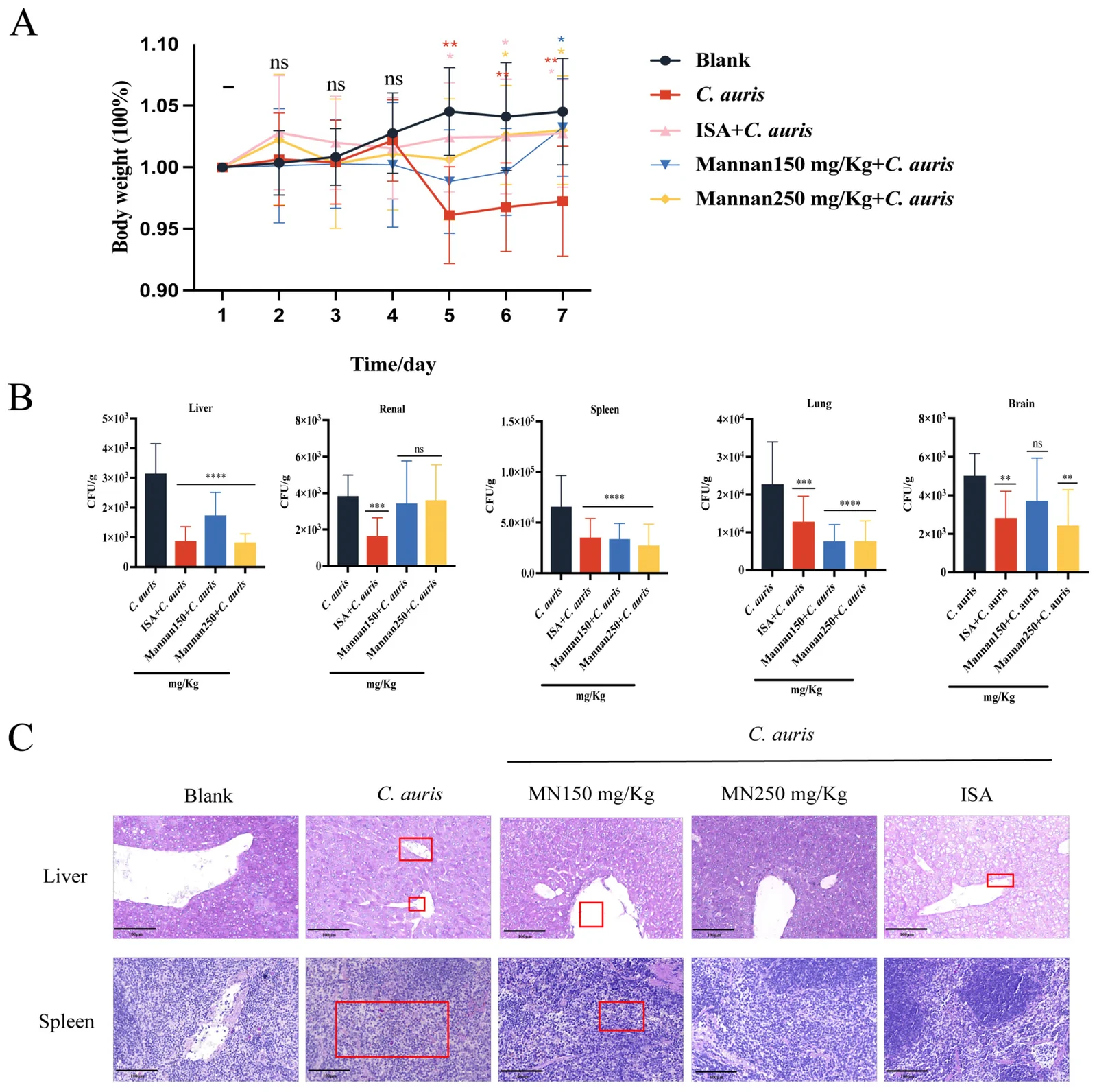

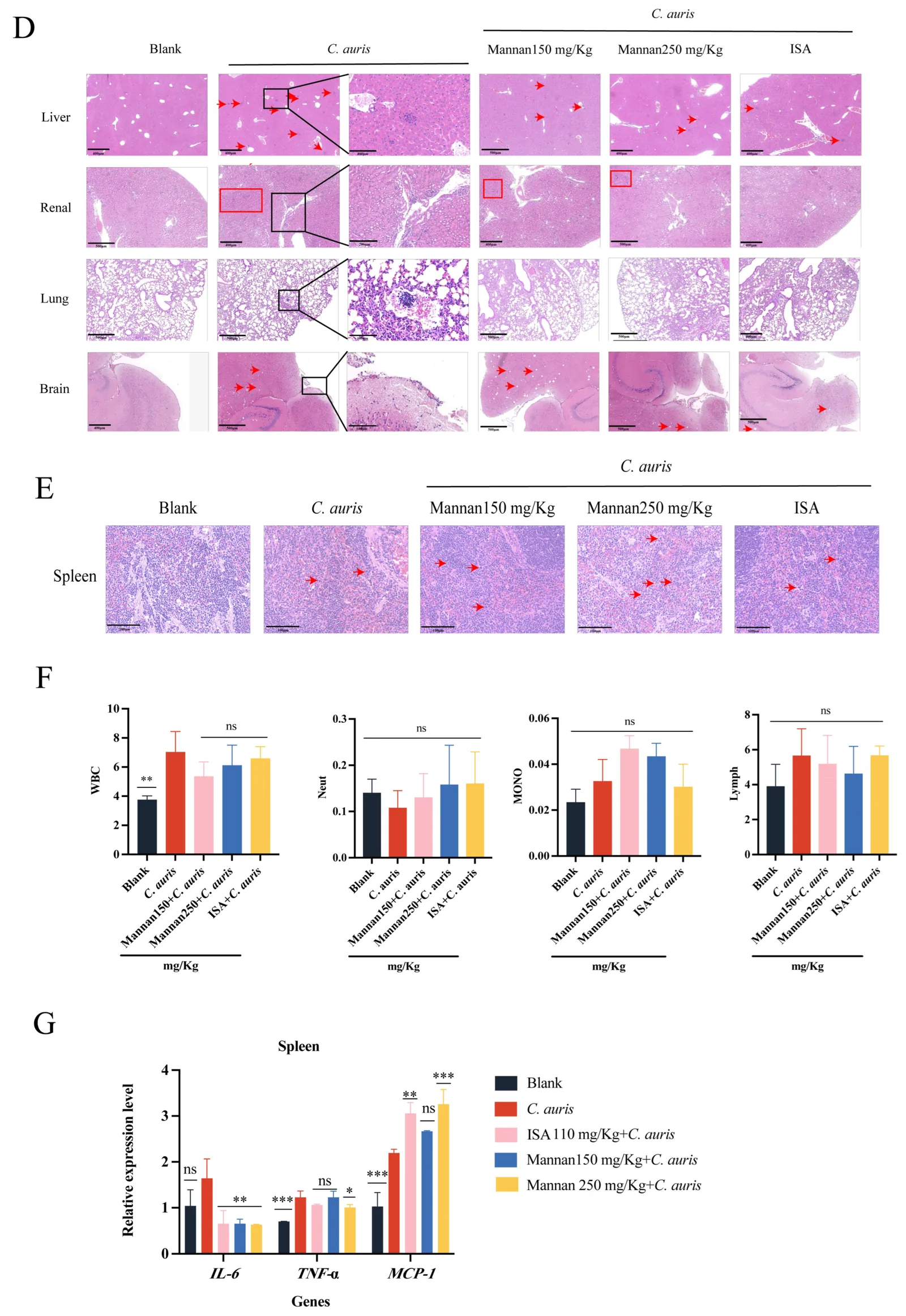
Effect of mannan in the prevention of *C. auris* in mice. The mice were prophylactically treated with mannan for 3 days, and blood was infected with *C. auris* for 3 days. (**A**) Mouse body weight changes; (**B**) colony count to detect fungal burden in liver, kidney, lung, brain and spleen tissues; (**C**) PAS staining of liver and spleen tissues; red boxes highlight regions with fungal colonization; (**D**) HE staining of liver, kidney, lung and brain tissues; black boxes mark magnified areas with obvious inflammatory cell infiltration. (**E**) HE staining of spleen tissue; arrows indicate infiltrated inflammatory cells; (**F**) a whole-blood cell analyzer was used to determine the peripheral blood immune cells in mice; (**G**) RT-qPCR was used to detect cytokine levels in mouse spleen tissue. For both (**F**) and (**G**), the control group and all prophylactic groups were compared with the *C. auris*-infected group. ns: indicates not significant; * indicates *p* < 0.05; ** indicates *p* < 0.01; *** indicates *p* < 0.001; ****: *p* < 0.0001.

To assess the protective effect of mannan against *C. auris* infection in vivo, a mouse bloodstream infection model was established and administered prophylactic treatment with mannan prior to infection. The results showed that mice in the mannan-treated group exhibited significantly less body weight loss after infection compared to the infected control group, followed by a gradual recovery in the later stages (Figure 4A), suggesting that mannan may alleviate infection-related systemic responses. Furthermore, tissue fungal burden analysis demonstrated that mannan treatment significantly reduced fungal loads in the liver, spleen, lungs, and brain tissues (Figure 4B). Of these, fungal clearance in the liver and spleen was most pronounced, indicating that mannan may enhance the antimicrobial capacity of key immune organs. PAS staining also revealed the presence of abundant fungus (purple-red granules indicated by red arrows) in the liver and spleen tissues of the *C. auris* group, whereas fungal load was markedly reduced in the mannan prophylaxis group (Figure 4C). H&E staining showed hepatocyte swelling and extensive inflammatory cell infiltration in the liver tissue of the *C. auris* group, while inflammation was significantly attenuated in the mannan-treated group (Figure 4D). In lung tissues, the *C. auris* group exhibited airway wall thickening and moderate inflammatory infiltration, whereas inflammation was milder in the mannan group. The overall brain tissue structure remained relatively intact; however, vacuolar degeneration was observed in the brain parenchyma of the *C. auris* group, with scattered inflammatory cell infiltration in the meninges. Mannan treatment partially alleviated inflammation but did not significantly improve vacuolar changes. No obvious necrosis was observed in the kidney tissue; however, multifocal inflammatory cell infiltration was present in the interstitium of the *C. auris* group, with no significant change following mannan treatment. Notably, a significant increase in macrophage numbers was observed in the spleen following mannan treatment (Figure 4E), suggesting that mannan may promote immune cell activation or recruitment, thereby enhancing local immune responses.

To further explore the immunomodulatory effects of mannan on immune cells, peripheral blood cell counts were analyzed (Figure 4F). Compared with the infected control group, the mannan prophylaxis group showed no significant changes in total white blood cells, neutrophils, or lymphocytes; however, monocyte counts exhibited an increasing trend. Moreover, cytokine analysis of spleen tissues showed that mannan prophylaxis effectively reduced expression of the pro-inflammatory cytokine IL-6 (Figure 4G). At the 250 mg/kg dose, mannan also significantly suppressed TNF-α expression. Conversely, mannan significantly upregulated the chemokine monocyte chemoattractant protein-1 (MCP-1) (Figure 4G). In summary, mannan demonstrated significant tissue protection, alleviation of inflammation, and immunoregulatory effects in the mouse model of *C. auris* infection, particularly by promoting macrophage recruitment and modulating inflammatory cytokine expression.

### 3.5. Immunomodulatory Effects of Mannan of Macrophage Recruitment in a Zebrafish Model of C. auris Infection

**Figure 5:**
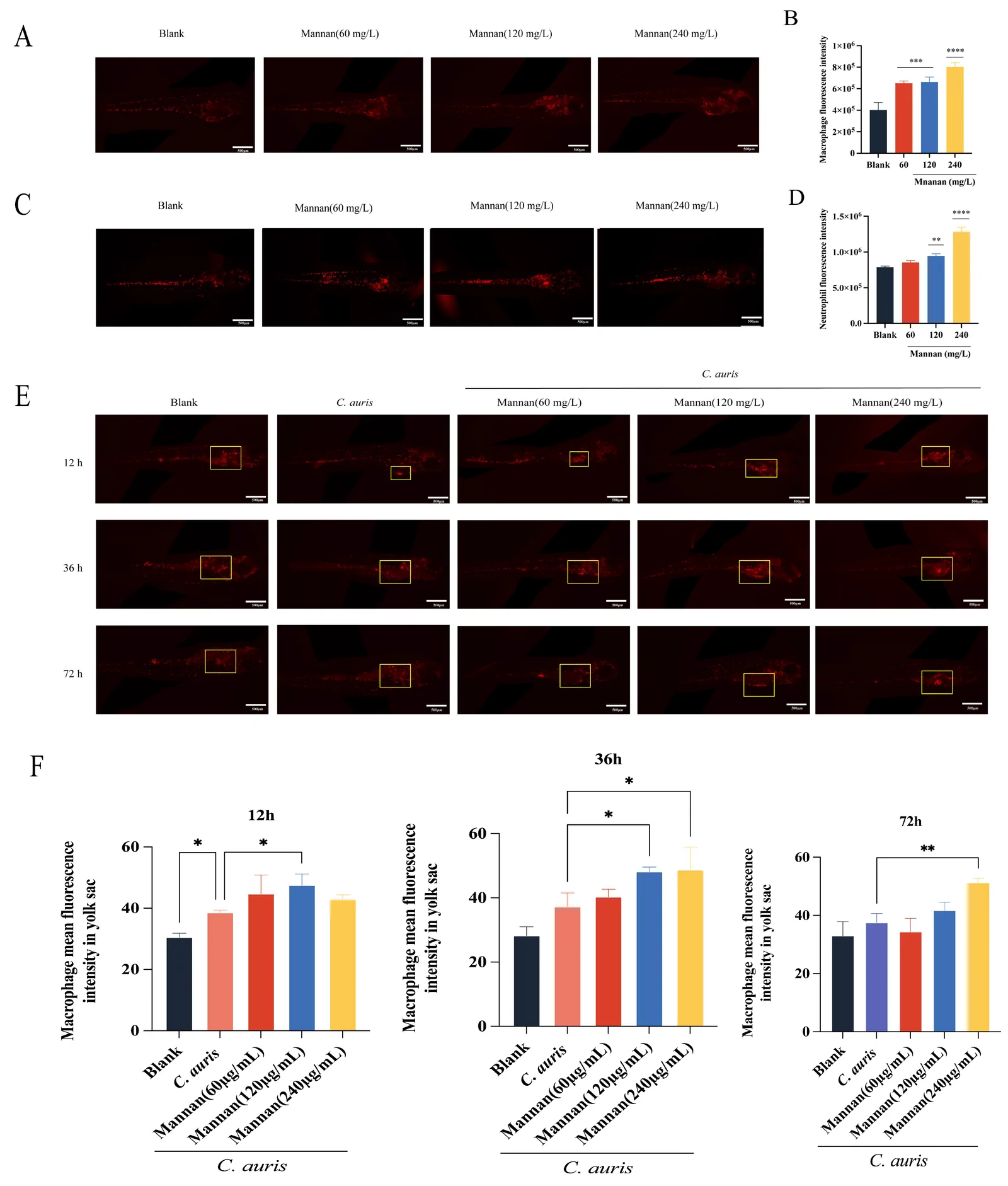

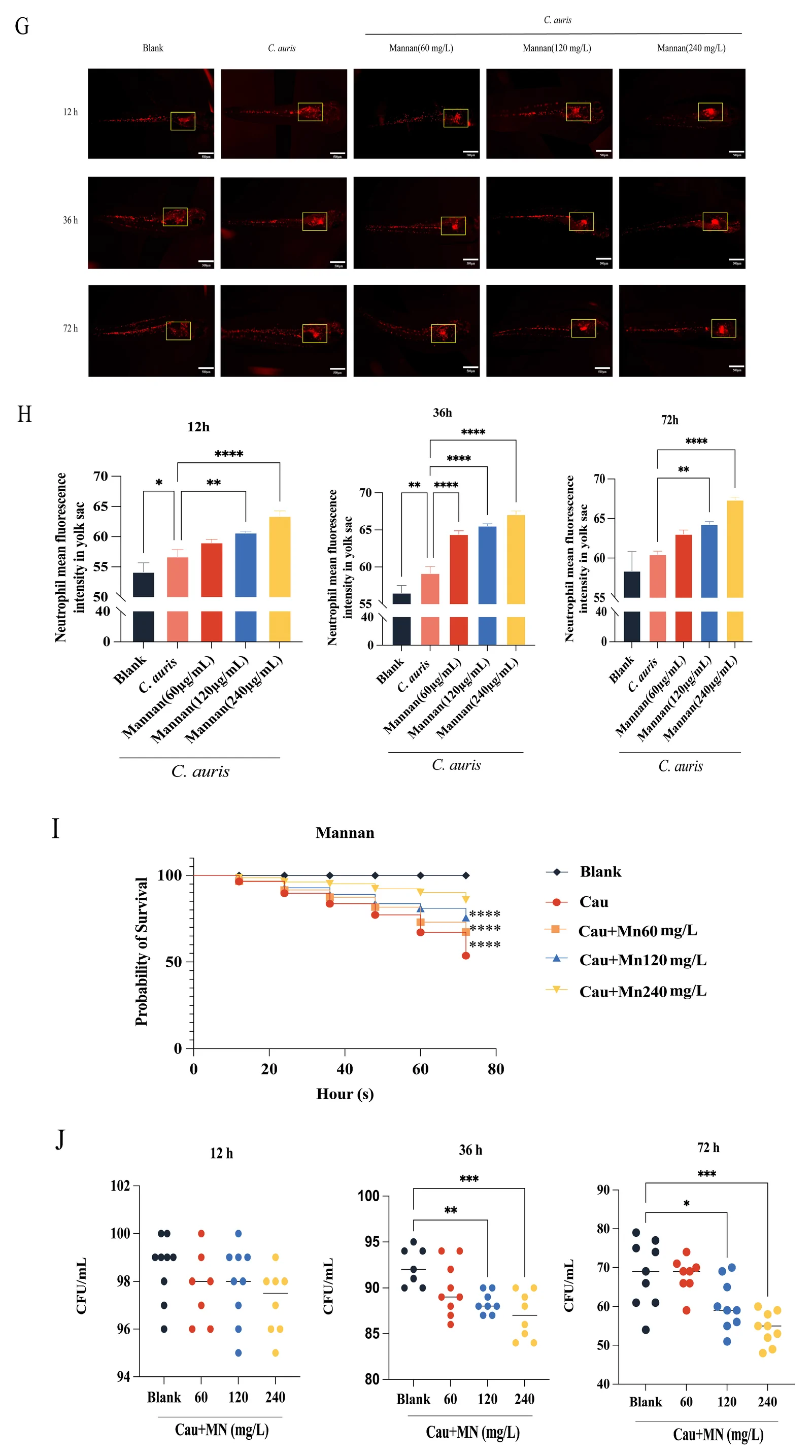
Effects of mannan on macrophage and neutrophil recruitment in zebrafish. Three-day-old zebrafish larvae were exposed to mannan at concentrations of 60, 120, or 240 mg/L for 24 h, followed by yolk sac injection with *C. auris*. The larvae were observed to assess toxicity and imaged at 12, 36, and 72 h post-infection. (**A**,**B**) Effects of mannan on the total number of macrophages in zebrafish. (**C**,**D**) Effects of mannan on the total number of neutrophils in zebrafish. (**E**,**F**) Effects of mannan on macrophage recruitment to the zebrafish yolk sac; the yellow boxes indicate the yolk sac region. (**G**,**H**) Effects of mannan on neutrophil recruitment to the zebrafish yolk sac; the yellow boxes indicate the yolk sac region. (**I**) Zebrafish survival at 72 h post-infection. (**J**) Fungal burden in zebrafish at the indicated time points post-infection. For statistical analyses, the control group and all prophylactic groups were compared with the *C. auris*-infected group. ns: indicates not significant; * indicates *p* < 0.05; ** indicates *p* < 0.01; *** indicates *p* < 0.001; ****: *p* < 0.0001.

The zebrafish model was used to systematically evaluate the immunomodulatory potential of mannan within the context of the innate immune system and its in vivo protective effect against *C. auris* infection. Early-stage zebrafish embryos possess only innate immunity, providing a simplified platform for determining the effects of immunomodulators on innate immune responses and helping clarify specific mechanisms [34]. First, the safety of mannan in zebrafish by evaluating morphological toxicity indicators under uninfected conditions was assessed. Zebrafish larvae at 72 hpf were exposed to mannan solutions at concentrations of 60, 120, and 240 mg/L for 24 h. No typical toxicity manifestations such as pericardial edema, spinal curvature, abnormal tail fin development, or visceral malformations were observed. With regard to immune cells, mannan had significant effects on the numbers of macrophages and neutrophils. Under uninfected conditions, all tested concentrations (60, 120, and 240 mg/L) significantly increased macrophage numbers (Figure 5A,B), while only 120 and 240 mg/L concentrations significantly elevated neutrophil numbers (Figure 5C,D).

To further evaluate the regulatory effects of mannan on immune cell migration and recruitment following *C. auris* infection, the zebrafish yolk sac infection model and observed immune cell dynamics at 12, 36, and 72 h post-infection was employed. The results showed that 60 mg/L mannan had no significant effect on macrophage recruitment, whereas 120 mg/L significantly enhanced macrophage migration at 12 h and 36 h, and 240 mg/L significantly increased macrophage recruitment at 72 h (Figure 5E,F). For neutrophils, only the 60 mg/L concentration significantly enhanced recruitment at 36 h post-infection (Figure 5G,H). Regarding anti-infective effects, zebrafish pretreated with mannan exhibited significantly increased survival rates (Figure 5I). Notably, fungal burden was significantly reduced at 36 h and 72 h post-infection in the 120 mg/L and 240 mg/L treatment groups (Figure 5J). In summary, the zebrafish experimental results demonstrate that mannan, with non-toxic conditions, can enhance the quantity and recruitment capacity of innate immune cells, thereby improving host survival and fungal clearance efficiency in *C. auris* infection.

## 4. Discussion

Similar to natural polysaccharides such as Chitosan and β-glucan reported in previous studies, mannan can enhance macrophage function by activating the innate immune response, suggesting a common mechanism of polysaccharides in immunomodulation [35,36,37]. Further investigations confirmed that it can activate downstream signaling pathways by binding to C-type lectin receptors (such as Dectin-2 and mannose receptors), thereby inducing macrophage activation and enhancing phagocytosis, inflammatory factor secretion and antigen presentation ability [38,39]. In this study, mannan significantly increased the ability of Ana-1 cells to phagocytose *C. auris*. Further functional experiments confirmed that it not only promotes pathogen recognition and uptake but also activates intracellular antifungal mechanisms. Moreover, mannan itself has no direct antifungal effect on *C. auris* in vitro; nevertheless, its enhanced bactericidal effect depends on the activation of host immune cells. It should be noted that *C. auris* exhibits morphological plasticity, including the formation of cell aggregates and pseudohyphae-like structures, which may differ in cell wall composition and host interactions. Therefore, although the yeast-form model used in this study provides a standardized system to evaluate the immunomodulatory effects of mannan, further studies are needed to investigate its effects on different morphological forms of *C. auris*. The clinical *C. auris* strain used in this study was isolated from a patient specimen and confirmed as *C. auris* in our previous study, and it was classified as Clade I [40]. Therefore, the findings of this study may be strain- or clade-specific and cannot be directly extrapolated to other *C. auris* clades. Further studies using strains from different clades are warranted to determine whether the observed immunomodulatory effects of mannan are broadly applicable across *C. auris*. Therefore, whether the observed immunomodulatory effects of mannan are conserved among different *C. auris* clades remains to be further investigated. In addition, the environmental adaptation of *C. auris* and related species may contribute to their pathogenic potential. Recent evidence suggests that interactions with environmental microorganisms, such as free-living amoebae, may promote the acquisition of virulence-associated traits in *Candidozyma* species, highlighting the importance of considering both host immunity and environmental evolution in understanding fungal pathogenicity [41].

The immunological activity of a polysaccharide is significantly affected by its structural characteristics, such as degree of branching, molecular weight, and recognition receptor type [42]. Therefore, an in-depth analysis of the receptor binding characteristics of mannan and its signal transduction mechanism is highly important. In this study, a total of 117 DEGs were screened via transcriptome sequencing analysis (Figure 2). Moreover, the inflammatory factors IL-6 and IL-1β and the chemokine CXCL2 were significantly upregulated following mannan stimulation, suggesting that they can amplify proinflammatory signals and promote immune cell recruitment and activation, thereby enhancing antifungal defense [43,44,45]. Although in the transcriptome data, the log_2_ fold change of GPCR was only 0.89 and did not meet the conventional threshold for differential significance, as a member of the GPCR family, it is widely involved in regulating macrophage inflammatory responses and phagocytic functions [46]. For example, GPCRs sense a variety of extracellular signals and transduce them into the cell, thereby modulating multiple physiological processes, including immune responses and anti-infective mechanisms [47,48]. Therefore, even modest changes in expression may trigger downstream signaling pathways through cascade amplification mechanisms. Molecular docking results revealed that mannan has a strong ability to bind to GPCRs (binding energy of −7.8 kcal/mol), supporting its potential as a functional receptor (Figure 3). However, molecular docking provides only computational evidence of potential molecular interactions and does not establish direct physical binding between mannan and GPCRs. Thus, these results should be considered supportive rather than definitive evidence for GPCR involvement. Further studies using confocal colocalization, fluorescence resonance energy transfer (FRET), surface plasmon resonance (SPR), or other biophysical approaches will be required to experimentally validate the interaction between mannan and candidate GPCRs. In addition, studies have shown that the PI3K–NF-κB pathway plays an important role in regulating the expression of inflammatory factors and chemokines and is a key pathway through which macrophages perform immune functions [49,50]. As shown by RT–qPCR and Western blot detection, the expression of Akt tended to decrease, In the context of enhanced PI3K and NF-κB signaling, this moderate reduction in Akt activation may serve as a regulatory mechanism to prevent sustained or excessive immune activation, while still contributing to the enhanced antifungal activity of macrophage.

Previous studies using murine models of *C. auris* bloodstream infection have consistently reported key phenotypic outcomes, including significant body weight loss, fungal colonization in critical organs, and alterations in immune cell populations [51]. In line with these findings, our study demonstrated that prophylactic treatment with mannan effectively alleviated body weight loss and reduced fungal burdens in the liver, lungs, brain, and spleen (Figure 4). Additionally, histopathological analysis revealed attenuated tissue injury in several organs following mannan treatment, although the protective effects varied among different tissues. Notably, mannan treatment did not completely reverse all pathological alterations, such as inflammatory cell infiltration in the renal interstitium and vacuolar degeneration in the brain parenchyma, indicating that its protective effects were partial rather than complete. Previous studies have shown that *C. auris* infection induces a pronounced innate immune response characterized by increased production of pro-inflammatory cytokines, including IL-6 and TNF-α, which contribute to antifungal defense but may also cause tissue injury when excessively produced [52]. In addition, MCP-1 (CCL2) is recognized as a key chemokine involved in the recruitment of monocytes and macrophages to sites of fungal infection [53]. Consistent with these findings, histopathological analysis in our study revealed reduced tissue damage in the mannan-treated group, accompanied by increased splenic phagocyte proliferation and elevated peripheral blood monocyte counts. Furthermore, mannan treatment reduced IL-6 and TNF-α levels while enhancing MCP-1 expression, suggesting that mannan may modulate the inflammatory response by balancing antifungal immune activation and excessive inflammation, thereby contributing to partial tissue protection and enhanced monocyte/macrophage recruitment. These findings further support the successful establishment of the infection model and highlight the immunomodulatory potential of mannan. Unlike traditional antifungal drugs such as amphotericin B and fluconazole, which directly target the pathogen, mannan’s protective effects are primarily mediated through activation of the monocyte–macrophage immune axis, offering a unique “immune activation–tissue protection” strategy against infection [54]. In comparison to existing reports on antifungal agents, which often emphasize fungal clearance and survival outcomes, our study provides a novel perspective by highlighting the role of immune activation in enhancing host defense mechanisms [55]. However, given the organ-dependent protective effects observed in our study, further investigations are required to clarify the optimal conditions under which mannan-mediated immune modulation can achieve broader tissue protection. This distinct immunomodulatory action represents a promising alternative approach to conventional antifungal therapies [56,57].

Zebrafish models, widely used for investigating systemic fungal infections and immune responses due to their rapid development, transparency, and robust innate immune system, were employed to further explore mannan’s immunoprotective effects [34]. In this study, we first assessed the developmental safety of mannan in zebrafish larvae, and found no evidence of morphological abnormalities or increased mortality at concentrations ranging from 60 to 240 mg/L (Figure 5). In terms of immune response, mannan treatment further enhanced macrophage recruitment to the infection site, with the most pronounced effect observed in the 240 mg/L group at 72 h post-infection. These findings align with our murine data, further validating mannan’s role in modulating innate immune responses [35,36,37].

## 5. Conclusions

**Figure 6:**
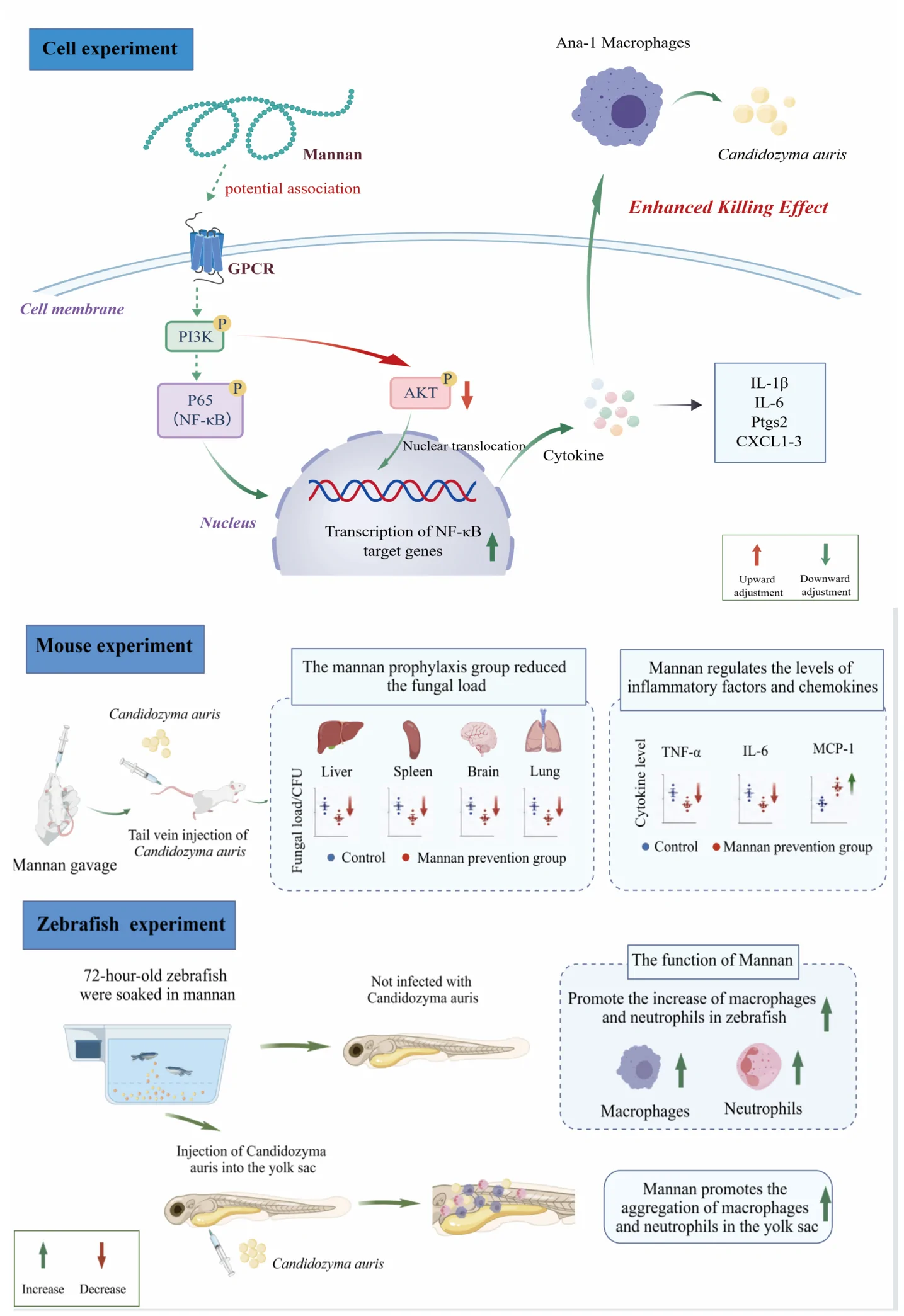
Proposed mechanism by which mannan enhances host antifungal immunity against *C. auris* in vitro and in vivo. In Ana-1 macrophages, mannan is associated with changes in the GPCR/PI3K/NF-κB signaling pathway, leading to increased phosphorylation of PI3K and NF-κB p65, while suppressing Akt phosphorylation. These signaling events are associated with the transcription of NF-κB target genes and enhance the antifungal activity of macrophages against *C. auris*. In the mouse model, prophylactic administration of mannan prior to *C. auris* infection significantly reduced fungal burdens in multiple organs, including the liver, spleen, brain, and lungs. Mannan treatment also modulated the host inflammatory response by decreasing the levels of pro-inflammatory cytokines (TNF-α and IL-6) while increasing the chemokine MCP-1. In the zebrafish model, mannan exposure increased the numbers of macrophages and neutrophils and promoted their recruitment and aggregation at the site of infection, thereby enhancing innate immune responses against *C. auris*. Collectively, these findings indicate that mannan improves host resistance to *C. auris* infection through activation of immune signaling pathways and enhancement of innate immune cell-mediated antifungal defenses.

In summary, this study systematically revealed the immunoprotective effect of mannan against *C. auris* infection in vitro and in vivo. Investigations revealed for the first time the potential mechanism by which mannan enhances the ability of mouse macrophages against *C. auris* infection, which is associated with the GPCR-PI3K-NF-κB signaling pathway (Figure 6). The in vivo immunoprotective effect also verified the potential of mannan as a novel antifungal immunomodulatory agent. These discoveries not only provide strong evidence for the application of mannan as a broad-spectrum immunomodulator in the prevention of systemic fungal infections but also provide a solid foundation for subsequent mechanistic research and clinical transformation. In the future, further more detailed investigations should be performed to elucidate the receptor recognition mechanism of mannan and expand it to other pathogen models.

## Data Availability

The original contributions presented in this study are included in the article. Further inquiries can be directed to the corresponding authors.

## Abbreviations

The following abbreviations are used in this manuscript:

Cau	*Candidozyma auris*
Ana-1	Mouse blood macrophages
YPD	Yeast extract peptone dextrose medium
CCK8	Cell Counting Kit-8
CFU	Colony-Forming Units
MN	Mannan
ISA	Isavuconazole
DMEM	Dulbecco’s Modified Eagle Medium
FBS	Fetal Bovine Serum
HE	Hematoxylin-Eosin staining
KEGG	Kyoto Encyclopedia of Genes and Genomes
PAS	Periodic Acid-Schiff stain
PAMPs	Pathogen-Associated Molecular Patterns
PRRs	Pattern Recognition Receptors
ROS	Reactive Oxygen Species
PBS	Phosphate-Buffered Saline
RT-qPCR	Real-time fluorescent quantitative PCR
TBST	Tris Buffered Saline with Tween
WB	Western Blot
NF-κB	Nuclear factor kappa-B

## Appendix A

**Table A1:** Primer sequence for RT-qPCR.

Gene	Primer Sequence (5′→3′)
*AKT*-F	CTACAACCAGGACCATGAGAAG
*AKT*-R	TCTTGAGCAGCCCTGAAAG
*GPCR*-F	CCTCACCTGGACACCATATAAC
*GPCR*-R	TGACGTAGCAAAGCCAGTAG
*PI3K*-F	CTGTGATGGGTAGAGGTTGATG
*PI3K*-R	GCTGCAGGTGTGTCTGATATT
*CXCL1*-F	GTGTCAACCACTGTGCTAGT
*CXCL1*-R	CACACATGTCCTCACCCTAATAC
*CXCL2*-F	GCCAAGGGTTGACTTCAAGAAC
*CXCL2*-R	GCTTCAGGGTCAAGGCAAACT
*CXCL3*-F	GCACCCAGACAGAAGTCATAG
*CXCL3*-R	ACTTGCCGCTCTTCAGTATC
*IL-6*-F	GTCTGTAGCTCATTCTGCTCTG
*IL-6*-R	GAAGGCAACTGGATGGAAGT
*IL-1β*-F	TCTGGGGAGGCACATCTTCT
*IL-1β*-R	CAGGTCCAAGTTGCCGTTTC
NF-κB p65-F	TTAAAAACCTGGATCGGAACCAA
NF-κB p65-R	GCATTAGCTTCAGATTTACGGGT
MCP-1-F	TCGCCGCTTAGTCACATACC
MCP-1-R	GGTCACCAGGTACACGTCAT
TNF-*α*-F	GGGCCTCAAAGGAAAGAATCT
TNF-*α*-R	GAGGTGCTGATGTACCAGTTGG
